# Limited Diagnostic Value of Cystoscopy in the Management of Gross Hematuria Due to Placenta Percreta: A Case Report

**DOI:** 10.7759/cureus.105666

**Published:** 2026-03-22

**Authors:** Majid Karimi, Mansoureh Zargar, Vahid Narouie, Taha Karimi

**Affiliations:** 1 Urology, Zahedan University of Medical Sciences, Zahedan, IRN; 2 Gynecology, Zahedan University of Medical Sciences, Zahedan, IRN; 3 Biological Sciences, University of California San Diego, San Diego, USA

**Keywords:** bladder invasion, cystoscopy, gross hematuria, placenta accreta spectrum, placenta percreta

## Abstract

Placenta percreta is the most severe form of abnormal placental invasion and is associated with significant maternal morbidity and mortality. Although bladder invasion may occur, gross hematuria is an uncommon and diagnostically challenging presentation. We report the case of a 35-year-old woman with a history of three prior cesarean sections who presented at 26 weeks of gestation with abdominal pain, spotting, acute urinary retention, and gross hematuria. Imaging suggested placenta previa with suspected placenta increta/percreta. Emergency cesarean delivery followed by hysterectomy was performed due to massive hemorrhage. Despite hysterectomy, persistent hematuria and hemodynamic instability continued. Cystoscopy revealed only nonspecific bladder wall hyperemia without an identifiable bleeding source. Definitive diagnosis and control of bleeding were achieved only after surgical re-exploration, which identified and ligated an actively bleeding intramural bladder vessel. This case highlights the limited diagnostic and therapeutic value of cystoscopy in placenta percreta-related hematuria. A normal or nonspecific cystoscopic examination should not exclude bladder invasion or delay surgical intervention in unstable patients.

## Introduction

Placenta accreta spectrum disorders are characterized by abnormal placental adherence to the uterine wall and are classified into three subtypes based on the depth of myometrial invasion. In placenta accreta, chorionic villi attach to the myometrium without invading it. In placenta increta, villi penetrate into the myometrium. In placenta percreta, the most severe form, placental tissue extends through the full thickness of the myometrium and uterine serosa, potentially invading adjacent organs, most commonly the urinary bladder [1,2]. This distinction carries significant clinical implications, as the depth of invasion directly determines the risk of life-threatening hemorrhage, the likelihood of organ involvement, and the complexity of surgical management. However, accurate preoperative differentiation between these subtypes remains challenging, as imaging findings can be subtle and overlapping, and a definitive diagnosis often requires intraoperative or histopathological confirmation.

The incidence of placenta accreta spectrum disorders has increased in recent decades, largely due to rising cesarean section rates [3]. Bladder invasion is associated with severe obstetric hemorrhage and urological complications [4,5]. Although vaginal bleeding is common, gross hematuria remains a rare presenting symptom, even in cases with confirmed bladder involvement [6,7].

We present a case of placenta percreta complicated by massive hematuria in which cystoscopy failed to identify the bleeding source, emphasizing the limitations of cystoscopy in this clinical scenario.

## Case presentation

A 35-year-old woman, gravida 5 para 3 (G5P3), with a history of three previous cesarean deliveries, presented at 26 weeks of gestation with abdominal pain, vaginal spotting, and acute urinary retention. Her medical history was significant for hypothyroidism, treated with levothyroxine.

On initial evaluation in the emergency department, the patient presented with gross hematuria and significant clot retention. Following bladder catheterization with a 22-French three-way Foley catheter, gross hematuria with bright red blood and clots was noted. Concurrently, vaginal spotting progressed to metrorrhagia, establishing that active hemorrhage from both uterine and vesical sources was present prior to any surgical intervention.

**Figure 1:**
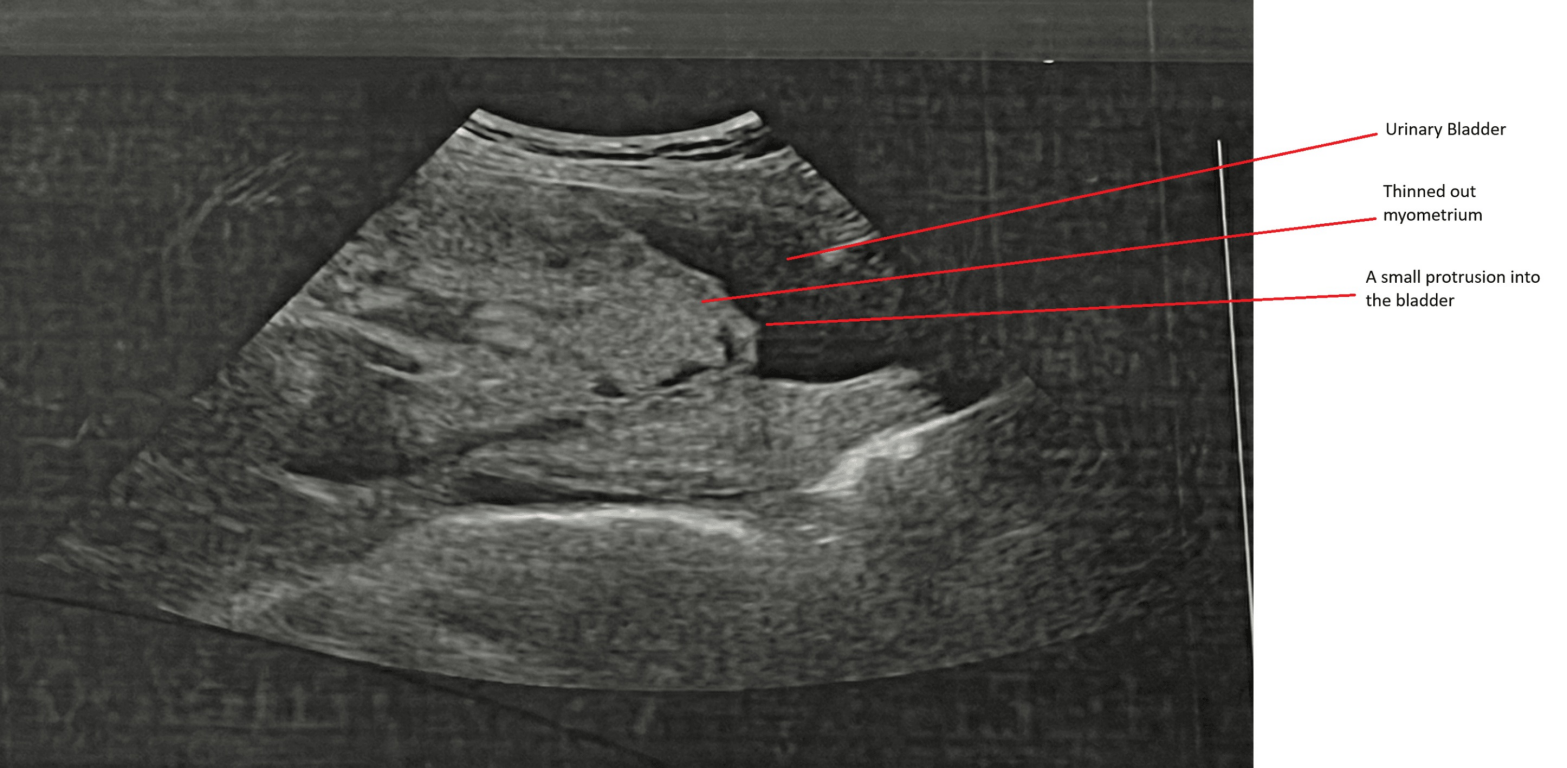
USG scan of the placenta. Obsteric USG demonstrating anterior placenta pervia with thinning of the myometrium and focal protrusion toward the urinary bladder (arrows), suggestive of placenta percreta. USG: ultrasonography.

Ultrasonography demonstrated an anterior placenta with complete placenta previa. Hypoechoic areas with internal vascular flow were observed, and the myometrial thickness between the placenta and bladder wall was absent in the lower uterine segment, raising suspicion for placenta increta or percreta (Figure 1).

Cross-sectional imaging with MRI was not performed due to the emergent clinical presentation and limited availability in this setting. CT was deferred given concerns regarding fetal radiation exposure.

Due to uterine contractions and hemodynamic concerns, an emergency cesarean section was performed. A male infant was delivered, after which massive intraoperative hemorrhage necessitated an immediate hysterectomy. During surgical exploration, placental tissue was observed extending beyond the uterine serosa and closely adherent to the posterior wall of the urinary bladder. The intraoperative findings suggested direct placental invasion toward the bladder wall. During the procedure, the patient required transfusion of five units of packed red blood cells and six units of fresh frozen plasma due to significant blood loss and concern for possible disseminated intravascular coagulation. Intraoperatively, placental extension beyond the uterus was noted, but no obvious bladder injury was identified. Methylene blue intravesical instillation confirmed bladder integrity [8].

Postoperatively, persistent gross hematuria continued despite continuous bladder irrigation and blood product transfusion. Cystoscopic evaluation demonstrated diffuse erythema and hyperemia of the posterior bladder wall without active bleeding or mucosal disruption. Given ongoing hematuria, tachycardia, and hypotension, re-exploration was undertaken.

**Figure 2:**
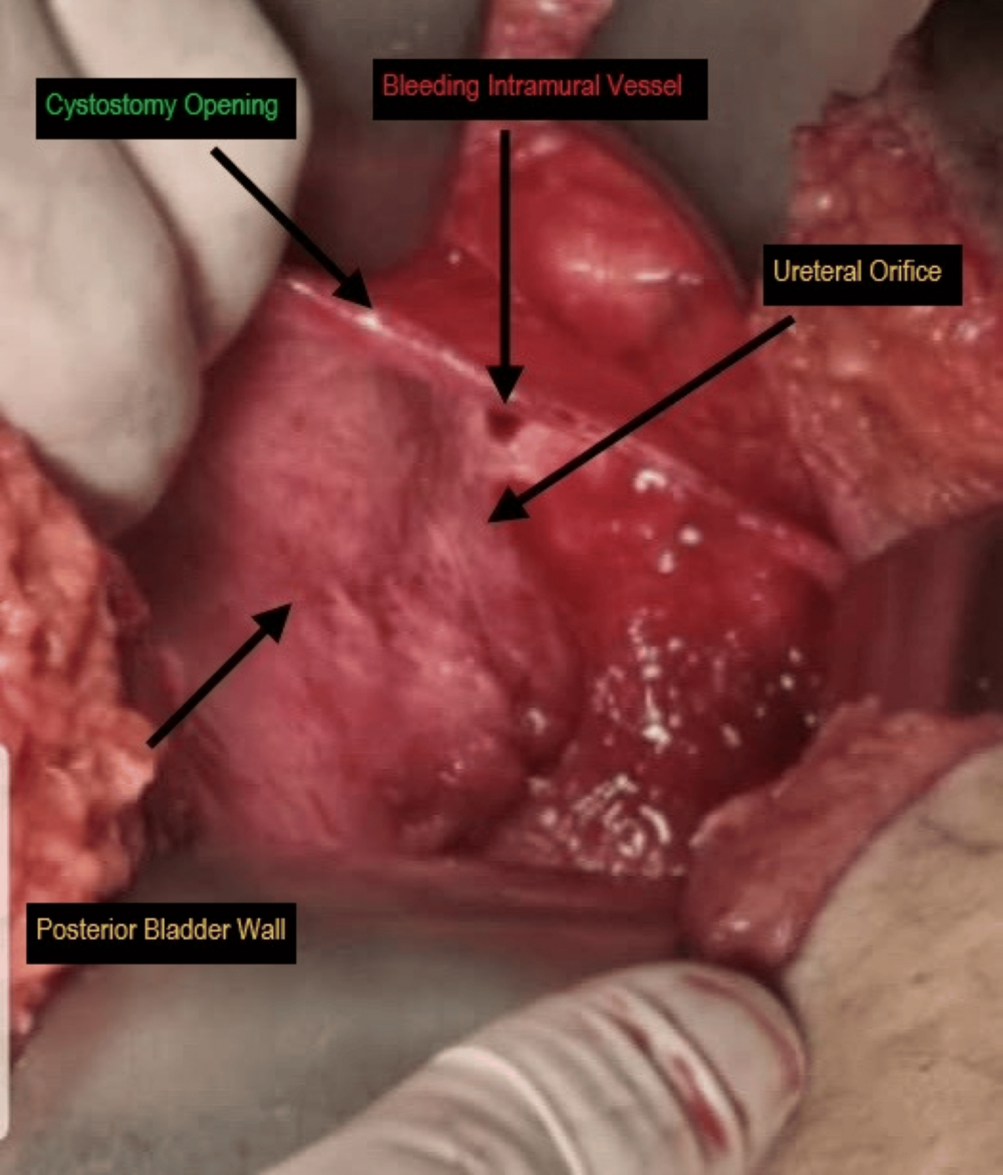
Bladder dome cystotomy with annotated surgical landmarks. Intraoperative view following cystotomy through the bladder dome. The cystotomy opening provides direct visualization of the posterior bladder wall. An actively bleeding intramural vessel is identified within the bladder wall, representing the source of persistent gross hematuria that was not visualized during prior cystoscopic evaluation due to its submucosal location. The ureteral orifice is identified in proximity to the bleeding site. The surrounding mucosal surface of the posterior bladder wall appears grossly intact, consistent with the nonspecific cystoscopic findings of diffuse erythema and hyperemia without mucosal disruption.

Upon cystotomy through the bladder dome, a large actively bleeding intramural vessel within the bladder wall was identified and ligated (Figure 2) [9]. Ureteral patency was confirmed. The bladder was repaired in two layers, and the patient was stabilized postoperatively. She was discharged one week later, and the Foley catheter was removed after two weeks without complications.

## Discussion

Gross hematuria during pregnancy is uncommon and presents a diagnostic challenge, particularly in patients with prior cesarean sections. Although placenta percreta may invade the bladder, hematuria is reported in fewer than one-third of such cases [10].

Cystoscopy is widely used as a first-line diagnostic tool for gross hematuria due to its accessibility and low morbidity. However, in placenta percreta, placental invasion often involves submucosal or intramural bladder vessels, sparing the mucosa [11]. Consequently, cystoscopy may demonstrate only nonspecific findings or appear normal, resulting in false reassurance. The pathophysiology of hematuria in this context involves placenta-driven neovascularization within the submucosal and intramural layers of the bladder wall. These pathological vessels, subjected to the increased blood flow of pregnancy, may erode through the mucosa via microscopic disruption sufficient to permit hemorrhage into the bladder lumen but below the threshold of cystoscopic detection. This mechanism is analogous to submucosal vascular malformations of the urinary tract, which are well documented to cause clinically significant hematuria despite a grossly intact mucosal surface.

Washecka and Behling reported that cystoscopy was diagnostically unhelpful in the majority of patients with placenta percreta and bladder invasion, even in cases with massive hematuria [10]. Our case supports this observation and underscores that negative cystoscopic findings do not exclude ongoing bladder hemorrhage [12].

An important diagnostic consideration in cases of placenta percreta with postoperative hematuria is the possibility of iatrogenic bladder injury during hysterectomy. In the present case, however, gross hematuria with clot retention was documented in the emergency department prior to surgical intervention, and vaginal spotting had progressed to metrorrhagia concurrently. The presence of active hemorrhage from both vesical and uterine sites before any operative procedure strongly supports a pathological mechanism driven by placental neovascularization rather than surgical trauma. Methylene blue instillation following hysterectomy further confirmed the absence of transmural bladder injury, reinforcing this interpretation.

Additionally, although coagulopathy including disseminated intravascular coagulation was considered as a contributing factor, the continued formation of organized clots and the localized nature of the hematuria favored a focal vascular source, a distinction that ultimately guided the decision for surgical re-exploration and led to the identification of the bleeding vessel.

Although CT angiography may have identified the active bleeding source and guided intervention, hemodynamic instability in this case precluded a safe transfer for imaging and necessitated immediate surgical decision-making. This underscores the importance of maintaining a low threshold for surgical exploration when cystoscopy fails to identify a bleeding source in the setting of suspected placenta percreta.

In hemodynamically unstable patients, reliance on cystoscopy may delay life-saving surgical intervention [13,14]. Early recognition, multidisciplinary coordination, and a low threshold for surgical exploration are critical in managing placenta percreta-related complications [15]. Cystoscopic images were not retained due to the urgency of the clinical setting and technical limitations of the equipment used during the emergent evaluation. Although histopathological confirmation is ideal, intraoperative findings combined with clinical and imaging features may strongly suggest placenta percreta in emergency settings. 

## Conclusions

Cystoscopy has limited diagnostic and therapeutic value in cases of placenta percreta with bladder involvement. A normal or nonspecific cystoscopic appearance should not exclude bladder invasion or delay surgical exploration in hemodynamically unstable patients. In pregnant women with prior cesarean sections presenting with gross hematuria, placenta percreta must be strongly considered, and prompt multidisciplinary management is essential to reduce maternal morbidity and mortality.
